# The co-occurrence of multiple sclerosis and Evans syndrome: A case report

**DOI:** 10.22088/cjim.11.3.343

**Published:** 2020-05

**Authors:** Saeideh Salehizadeh, Abdorreza Naser Moghadasi, Mohammad Ali Sahrain

**Affiliations:** 1 Multiple Sclerosis Research Center, Neuroscience Institute, Tehran University of Medical Sciences, Tehran, Iran

**Keywords:** Multiple sclerosis, Evans syndrome, Autoimmune disorders

## Abstract

**Background:**

Evans syndrome is an uncommon autoimmune disorder manifested by fatigue, jaundice, pallor, purpura and petechiae. The main characteristics of this rare disease are simultaneous or sequential existence of positive anti-globulin test, immune thrombocytopenia (ITP) and autoimmune hemolytic anemia (AIHA). Evans syndrome as an autoimmune disorder can be associated with other diseases. The concomitancy of Evans syndrome and multiple sclerosis (MS) has not been reported so far. In this case study, a -21-year old male with concomitant Evans syndrome and MS has been reported.

**Case Presentation:**

A 21-year-old male of Iranian origin and known case of Evans syndrome, was referred to our hospital for better evaluation. Evans syndrome was presented with acute jaundice, purpura, petechiae, and easy bruising when he was 9.He was under treatment of corticosteroid and cytotoxic agents, and presented with left lower extremity paresis for 5 months before admission to our hospital. According to neuroimaging and pathologic results, multiple sclerosis (MS) was diagnosed. Hence, we decided to treat the patient with rituximab. The patient has been stable without any further exacerbation or increase in disability progression after 2 years from diagnosis.

**Conclusion:**

Evans syndrome can be associated with other autoimmune disorders. For our case, we have reported this association with MS.

Multiple sclerosis (MS) is recognized as a demyelinating disease of the central nervous system through autoimmune involvement of the brain and spinal cord (1-4). Evans syndrome is an uncommon autoimmune disorder. The main characteristics of this rare disease are simultaneous or sequential existence of positive anti-globulin test, immune thrombocytopenia (ITP) and autoimmune hemolytic anemia (AIHA) (5). For the first time, this rare syndrome was explained in 1951 (6). MS as an immune-mediated disorder can be associated with other autoimmune diseases. The prevalence of this association has not been sufficiently studied. However, several different autoimmune disorders have been reported in association with MS (7). Although coexistence of multiple sclerosis and autoimmune disease of thyroid gland, sjogren disease, type 1 diabetes, celiac disease, inflammatory bowel disease, vitiligo, psoriasis and other diseases has been reported in literature, only few cases of Evans syndrome with neurologic presentation exists (8 , 9). 

## Case presentation

A 21-year-old male of Iranian origin and known case of Evans syndrome, was referred to our hospital for better evaluation at September 2016. Evans syndrome was presented with acute jaundice, purpura, petechiae, and easy bruising when he was 9. At that time, clinical features accompanied by laboratory data manifestation of hemolytic anemia with thrombocytopenia led to diagnosis of Evans syndrome.

Hence, glucocorticoids were prescribed which dramatically improved his condition. He has been under cytotoxic therapy. Since then up to the admission time, azathioprine, cyclosporin, and splenectomy were done in the course of disease. Since then, he has been stable and no problem has been reported so far. His current illness started about 5 months ago when he complained of left lower extremity paresis. No complaints of diplopia, blurred vision, headache, vertigo and sphincter functions were reported. 

Habit history and family history were negative. His systemic examination was unremarkable except of abdominal striae secondary to steroid consumption and surgical scar of splenectomy. In neurologic examination, his strength was graded 4/5 both proximally and distally in the left lower extremity. Furthermore, there was hyperreflexia of both legs with bilateral Babinski sign. The remainder neurologic examination was normal.

**Figure 1:**
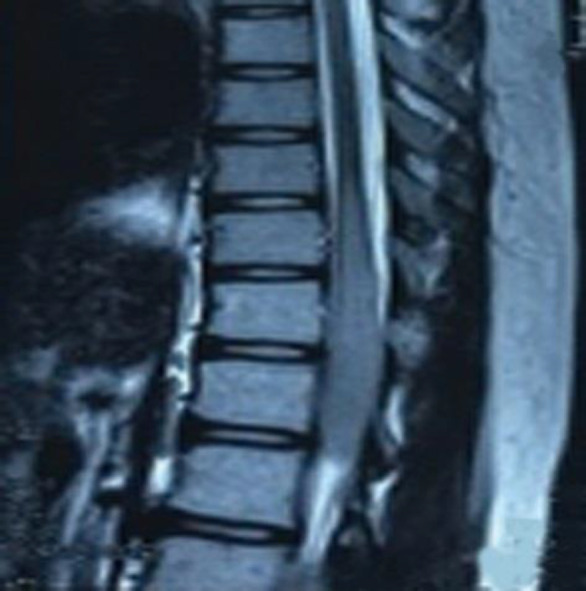
Thoracolumbar MRI revealed ill-defined intramedullary lesion

Spinal magnetic resonance imaging (MRI) showed T9 to L1 levels ill-defined intramedullary mass lesion. Cervical MRI was normal (fig 1). Unfortunately, brain MRI had not been performed.

**Figure 2:**
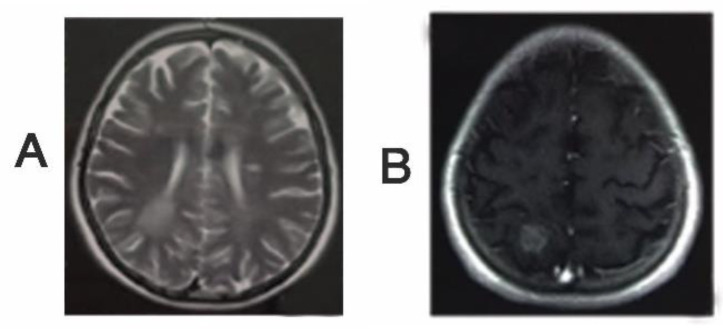
A, B: Brain MRI revealed high signal periventricular lesions. A tumefactive demyelinating lesion in right parietal lobe with surrounding vasogenic edema was also observed. There was post contrast enhancement of lesion

Based on these results, he went on surgery with astrocytoma and ependymal impression 1 month before referral to our clinic. During surgery, the neurosurgeon suspected misdiagnosis of disease according to appearance of lesion and took biopsy and did not resect the lesion. The specimen was evaluated which revealed glial tissue with reactive astrocytes and scattered parenchymal lymphocytes and macrophages as well as prominent perivascular cuffs of lymphocytes. The pathologist reported that tumefactive demyelinating disorders and peritumoral inflammatory reaction should be considered. Unfortunately, there was no accessibility to microscopic slides of lesions. Based on history and physical examination and pathology result, we asked for brain MRI with and without contrast where multiple high lesions in periventricular white matter were seen. Some of these had perpendicular axis in relation to lateral ventricle, reminding MS. A tumefactive demyelinating lesion in right parietal lobe with surrounding vasogenic edema was also observed. There was post contrast enhancement of lesion (fig. 2).

Biochemistry tests were normal. Also, vasculitis tests, angiotensin-converting enzyme (ACE) and aquaporin 4 antibody were negative. Based on our assessment and reevaluation, the diagnosis was MS concomitant with Evans syndrome. So, we decided to treat the patient with rituximab, as he suffered from two immune mediated disorders. He has been stable without any further exacerbation or increase in disability progression after 2 years from diagnosis.

## Discussion

Evans syndrome is a rare autoimmune disease which is mediated by auto antibodies, and has a chronic and relapsing nature. Only few cases of Evans syndrome concomitant with neurologic symptoms can be found in the literature. The case which has already been reported is manifested by severe aplastic anemia. Bone marrow transplantation was performed for patient. After that, the patient developed a mixed autoimmune syndrome including transverse myelitis, Evans syndrome, eosinophilia, pulmonary infiltrates, muscle pains and cramps, and dermatitis. Evans syndrome should be considered as a complication of graft-versus-host disease (GVHD) in this case (8). In other article, the neurological complications of Evans syndrome in children have been studied. Only eight patients had neurological complications including vertigo, myelitis, seizure, sensory neuropathy, intracranial hypertension and cranial nerve palsy (9).

The co-occurrence of MS and another autoimmune disease has been of substantial interest. The autoimmune mechanisms of these diseases are thought to have a major role in this concomitancy. The results of different studies speak for an increased coincidence of MS with other immunological disorders (10). Meanwhile, Evans is also an autoimmune disorder. Hence, the association of Evans and MS is probable.

In this patient, according to MRI and pathology results, co-existence of MS and Evans is possible. 

In conclusion MS can be associated with other autoimmune disorders such as psoriasis, uveitis, etc. as evidenced by existence of many articles regarding this issue. Accordingly, this time, we have reported this association with Evans syndrome. In the treatment of patients, these kinds of associations should be considered.

## References

[1] Galetta KM, Bhattacharyya S (2019). Multiple sclerosis and autoimmune neurology of the central nervous system. Med Clin North Am.

[2] Maitin IB, Cruz E (2018). Special considerations and assessment in patients with multiple sclerosis. Phys Med Rehabil Clin N Am.

[3] Presta I, Vismara M, Novellino F (2018). Innate immunity cells and the neurovascular unit. Int J Mol Sci.

[4] Lazibat I, Rubinić Majdak M, Županić S (2018). Multiple sclerosis: new aspects of immunopathogenesis. Acta Clin Croat.

[5] Rose NR, Mackay LR (2014). The autoimmune diseases. 5th ed.  USA: Elsevier.

[6] Jaime Pérez JC, Guerra-Leal LN, López-Razo ON, Méndez-Ramírez N, Gómez-Almaguer D (2015). Experience with Evans syndrome in an academic reference center. Braz J Hematol Hemotherapy.

[7] Sahraian MA, Owji M, Naser Moghadasi A (2016). Concomitant multiple sclerosis and another autoimmune disease: Does the clinical course change?. Clin Neurol Neurosurg.

[8] Richard S, Fruchtman S, Scigliano E (2000). An immunological syndrome featuring transverse myelitis, Evans syndrome and pulmonary infiltrates after unrelated bone marrow transplant in a patient with severe aplastic anemia. Bone Marrow Transplant.

[9] Pincez T, Neven B, Le Pointe HD (2019). Neurological involvement in childhood evans syndrome. J Clin Immunol.

[10] Belniak E, Stelmasiak Z, Papuć E (2007). Multiple sclerosis and other autoimmune diseases. Neurol Neurochir Pol.

